# The composition of lower genital tract microbiota correlates with *in vitro* fertilization and frozen embryo transfer outcomes in women with polycystic ovarian syndrome

**DOI:** 10.3389/fcimb.2025.1617187

**Published:** 2025-12-08

**Authors:** Yaoyao Tu, Zhiyang Zhou, Ye Lu, Bin Wei, Yingzhou Ge, Guolian Ding, Xi Dong, Jianzhong Sheng, Yixiang Zhang, Li Jin, Hefeng Huang

**Affiliations:** 1 Obstetrics & Gynecology Hospital of Fudan University, Shanghai Key Lab of Reproduction and Development, Shanghai Key Lab of Female Reproductive Endocrine Related Diseases, Shanghai, China; 2 Research Units of Embryo Original Diseases, Chinese Academy of Medical Sciences, Shanghai, China; 3 Department of Obstetrics and Gynecology, International Peace Maternity and Child Health Hospital, Shanghai Jiao Tong University, Shanghai, China; 4 Reproductive Medicine Center, Zhongshan Hospital, Fudan University, Shanghai, China; 5 Key Laboratory of Reproductive Genetics (Ministry of Education), Department of Reproductive Endocrinology, Women’s Hospital, Zhejiang University School of Medicine, Hangzhou, China; 6 Department of Obstetrics and Gynecology, The Fourth Affiliated Hospital, International Institutes of Medicine, Zhejiang University School of Medicine, Hangzhou, China; 7 Chinese Academy of Sciences (CAS) Center for Excellence in Molecular Plant Sciences, Shanghai Institutes for Biological Sciences (SIBS), Chinese Academy of Sciences, Shanghai, China; 8 University of Chinese Academy of Sciences, Shanghai, China; 9 Shanghai Key Laboratory of Embryo Original Diseases, Shanghai, China

**Keywords:** lower genital tract, microbiota, lactobacillus, polycystic ovary syndrome, *in vitro* fertilization and frozen embryo transfer

## Abstract

Adverse reproductive outcomes remain a significant concern for women of reproductive age with polycystic ovary syndrome (PCOS), yet the role of the lower genital tract (LGT) microenvironment has been largely overlooked. This study aimed to investigate the association between the LGT microbiome and the outcomes of *in vitro* fertilization and frozen embryo transfer (IVF-FET) in women with PCOS. A total of 191 reproductive-aged women undergoing assisted reproductive technology (ART) treatment between December 2018 and October 2021 were recruited. The LGT microbiota was profiled using 16S rRNA sequencing and analyzed in relation to ART outcomes and clinical parameters. Furthermore, cervical transcriptome sequencing was performed in a subset of PCOS patients to investigate whether LGT microbiota alterations were associated with functional changes in mucosal epithelial cells. The results demonstrate significant dysbiosis of the LGT microbiome in patients with PCOS, characterized by a reduction in *Lactobacillus* abundance. Among 72 PCOS patients undergoing IVF-FET, those with a relative *Lactobacillus* abundance of ≥50% (n = 57) exhibited significantly improved reproductive outcomes compared to those with *Lactobacillus* abundance <50% (n = 15). Elevated testosterone levels were identified as the most significant factor associated with a reduced abundance of *Lactobacillus* in PCOS patients. Transcriptomic analysis further revealed that the LGT microbiota was associated with maintaining mucosal epithelial barrier integrity and immune homeostasis in PCOS. In conclusion, the findings highlight that dysbiosis of the LGT microbiota may significantly influence reproductive outcomes in PCOS patients, emphasizing the importance of targeting the LGT microenvironment to improve ART success rates.

## 1 Introduction

The lower genital tract (LGT) microbiome constitutes a highly complex microbial ecosystem comprising millions of microorganisms and has garnered significant research interest in recent years. Beyond mere colonization, the LGT microbiome plays a pivotal role in regulating physiological processes that influence reproductive health and overall systemic homeostasis (Abou Chacra and Fenollar, 2021; Auriemma et al., 2021; Graham et al., 2021). In particular, *Lactobacillus* species are essential for maintaining vaginal health by preserving an acidic pH and suppressing the growth of pathogenic microorganisms (Mei and Li, 2022; Colella et al., 2023). Conversely, microbial dysbiosis-characterized by a depletion of *Lactobacillus* populations and an overgrowth of pathogenic bacteria-has been implicated in various reproductive health disorders, including bacterial vaginosis and sexually transmitted infections (Torcia, 2019). Furthermore, alterations in LGT microbiome composition due to dysbiosis have been associated with adverse reproductive outcomes, such as endometritis, infertility, pregnancy loss, and preterm labor (Elovitz et al., 2019; Peelen et al., 2019; Fu et al., 2020).

Polycystic ovary syndrome (PCOS) is the most prevalent endocrine disorder among women, affecting approximately 5-20% of reproductive-aged women worldwide. It is associated with a range of systemic complications, including reproductive dysfunction (Patel, 2018; Hoeger et al., 2021), disturbances in glucose metabolism, and an increased risk of cardiovascular disease (Liao et al., 2021; Wang et al., 2022). Women with PCOS are particularly susceptible to adverse pregnancy outcomes, such as pregnancy loss, gestational diabetes, preeclampsia, and preterm birth. Several risk factors have been identified, including hyperandrogenism, elevated luteinizing hormone (LH) levels, obesity, and insulin resistance (Liao et al., 2021). Our previous study revealed significant alterations in the LGT microbiota of women with PCOS compared to healthy reproductive-aged women (Tu et al., 2020). Unlike the well-established factors that are known to influence the reproductive outcomes of PCOS patients, the potential impact of the LGT microbiota remains underexplored. Moreover, the mechanisms regulating microbial composition in the LGT of women with PCOS, as well as the extent to which these microbial alterations affect local cellular function and reproductive health, remain poorly understood.

The study aimed to investigate the relationship between the LGT microbiome and *in vitro* fertilization–frozen embryo transfer (IVF-FET) outcomes in women with PCOS, and to explore the potential functional impact of microbial alterations on cervical epithelial cells.

## 2 Materials and methods

### 2.1 Sample collection and study design

This study recruited 191 women of reproductive age who sought assisted reproductive technology (ART) treatment at the International Peace Maternity and Child Health Hospital, the Obstetrics and Gynecology Hospital of Fudan University, and Zhongshan Hospital of Fudan University between December 2018 and October 2021. Participants were stratified into two groups: the PCOS group and the control group comprising women with male factor infertility. PCOS was diagnosed based on the Rotterdam criteria, which require the presence of at least two of the following three clinical characteristics: 1) oligo- or anovulation manifesting as menstrual irregularities (oligomenorrhea or amenorrhea), 2) ultrasound-confirmed polycystic ovarian morphology, and 3) clinical or biochemical evidence of hyperandrogenism. Individuals with Cushing’s syndrome, congenital adrenal hyperplasia, thyroid dysfunction, hyperprolactinemia, or androgen-secreting tumors were excluded from the study.

Prior to the collection of vaginal and cervical specimens, participants were confirmed to be non-pregnant, non-lactating, and not menstruating. They were also required to meet additional eligibility criteria: no antibiotic use within the preceding seven days, no cervical treatment or lavage within the past five days, and no sexual activity within 48 hours prior to sampling. Both the PCOS and control groups received hormone replacement therapy as part of their IVF-FET treatment protocol. Clinical outcomes of IVF-FET were categorized as follows: implantation success was defined as a serum human chorionic gonadotropin (hCG) level of ≥5 mIU/mL on Day 14; clinical pregnancy was defined as the presence of a visible gestational sac on ultrasound at Day 28; and live birth was defined as the delivery of a viable neonate at approximately Day 280.

### 2.2 Ethical approval

Written informed consent was obtained from all participants. This study was approved by the Institutional Review Boards of the International Peace Maternity and Child Health Hospital (Ethical Approval No. GKLW2018-10), the Obstetrics and Gynecology Hospital of Fudan University (Ethical Approval No. JIAI E2020-017), and Zhongshan Hospital of Fudan University (Ethical Approval No. B2021-326R).

### 2.3 Illumina sequencing and analysis of 16S rRNA

Vaginal and cervical specimens were collected using sterile cotton swabs and immediately transferred into sterile 2 mL DNA LoBind tubes containing physiological saline. The samples were then stored at -80 °C within 2 hours of collection. Genomic DNA was extracted using the QIAGEN PowerSoil Kit (Cat. No. 12888, QIAGEN) following the manufacturer’s instructions. The quality and integrity of the extracted DNA were assessed using a NanoDrop spectrophotometer (Thermo Scientific) and agarose gel electrophoresis. The 16S rRNA gene was amplified using primers targeting the V3–V4 hypervariable regions. The purified amplicons were used for library construction and subsequently sequenced on an Illumina HiSeq 2500 platform (San Diego, CA, USA). Negative controls, positive controls, and technical replicates were included throughout the process to monitor potential contamination and ensure reproducibility. Sequencing data were filtered to retain high-quality reads with a Phred score ≥ Q30. The resulting sequences were clustered into operational taxonomic units (OTUs) at a 3% dissimilarity threshold (i.e., 97% similarity). OTUs containing two or fewer sequences were excluded to minimize potential sequencing artifacts. The representative sequences of the remaining OTUs were taxonomically classified using the SILVA database. A total of 18,902 OTUs were identified, of which 93.6% were successfully assigned to a taxonomic classification.

Alpha diversity was assessed using indices such as Shannon’s index and Chao1, which quantify microbial richness and evenness. Beta diversity was evaluated using Bray–Curtis dissimilarity and visualized via principal coordinate analysis (PCoA) to characterize community structure and compare microbial compositions across groups. To ensure robust comparisons, confounders such as age, BMI, and other clinical variables were adjusted. Since the composition of the LGT microbiota may be affected by diverse clinical factors, spearman correlation analysis and Redundancy analysis (RDA) were further conducted to investigate the associations between clinical variables and microbial community structure.

### 2.4 RNA sequencing and analysis

The samples were collected, flash-frozen in liquid nitrogen, and stored at -80 °C until RNA extraction using TRIzol reagent. Total RNA was isolated, and cDNA libraries were constructed following standard protocols. These libraries were subsequently sequenced on the Illumina HiSeq 4000 platform at Genergy Ltd. (Shanghai, China). Raw data underwent quality filtering to remove low-quality reads and were subsequently aligned to the reference genome. Gene expression levels were quantified as Transcripts Per Million (TPM). Differential expression analysis and Gene Ontology (GO) enrichment analysis were performed using R (version 4.0.1) with relevant bioinformatics packages.

### 2.5 Statistical analyses

Reproductive outcomes of IVF-FET in PCOS patients stratified by LGT *Lactobacillus* content were compared using Fisher’s exact test. The relationships among dominant microbial genera in the LGT microbiota were assessed using Spearman’s correlation analysis. An unpaired Student’s *t*-test was used to compare continuous variables between the two groups, while a chi-square test was employed for the analysis of categorical variables. For GO enrichment analysis, Fisher’s exact test was applied to evaluate the significance of enrichment across biological process, cellular component, and molecular function categories. All statistical analyses were conducted using R software (version 4.0.1), with a *p*-value ≤ 0.05 considered statistically significant.

## 3 Result

### 3.1 LGT microbiota remains relatively stable throughout the menstrual cycle

**Figure 1:**
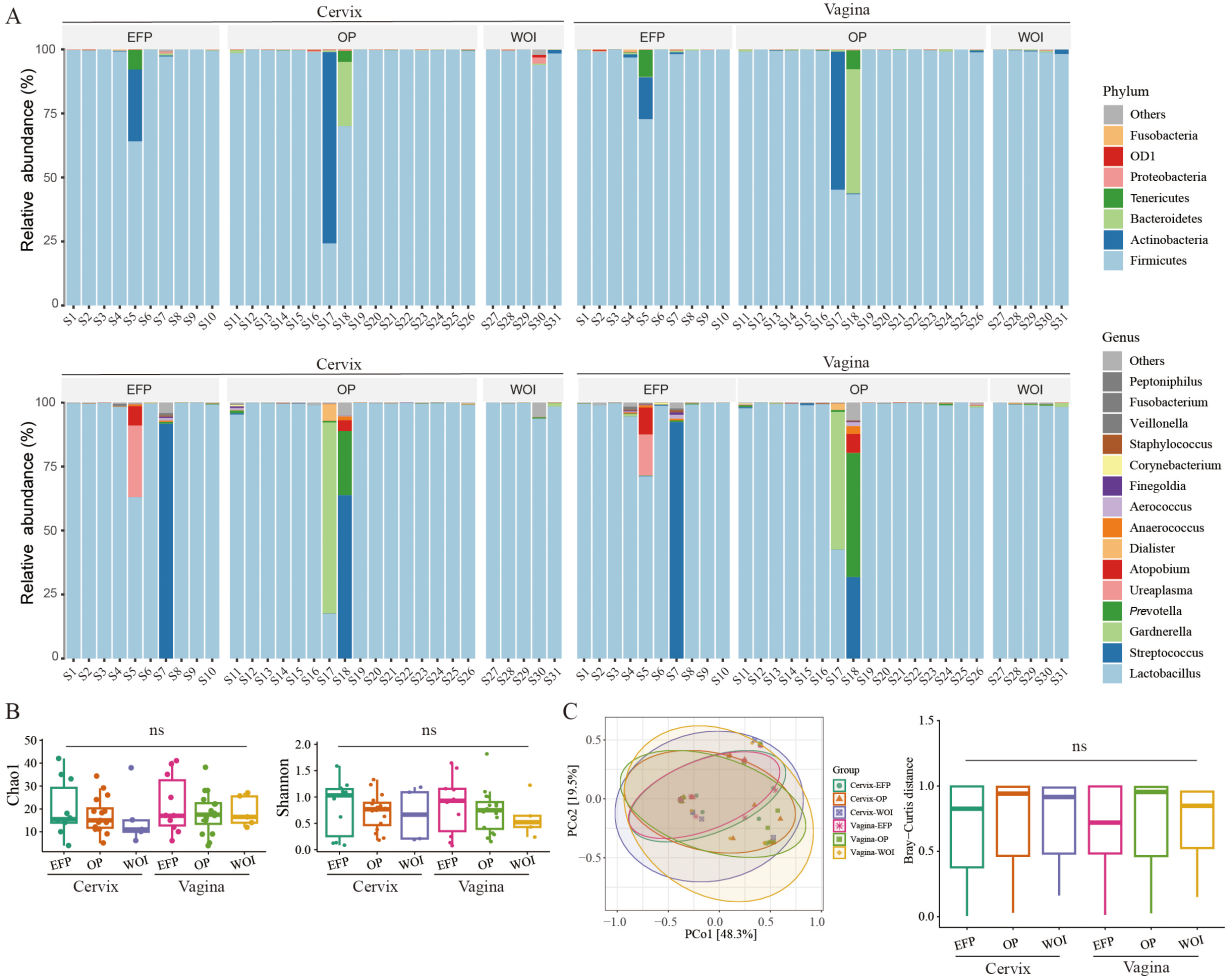
LGT microbiota stays stable across different phases of the menstrual cycle. **(A)** Microbial composition at the phylum (top) and genus (bottom) levels in cervical and vaginal samples collected during the early follicular phase (EFP), ovulatory phase (OP), and window of implantation (WOI). Each bar represents an individual sample; **(B)** Alpha diversity of the LGT microbiota in cervical and vaginal samples across menstrual phases, assessed using Chao1 and Shannon indices; **(C)** Beta diversity of the LGT microbiota in cervical and vaginal samples across menstrual phases, performed using principal coordinate analysis (PCoA) based on Bray-Curtis distances; ns, not significant.

A total of 62 specimens (31 vaginal and 31 cervical swabs) were collected from 22 healthy women of reproductive age with regular menstrual cycles at three distinct stages: the early follicular phase (EFP, within 3 days after menstruation), the ovulation phase (OP, 1 day before or after ovulation), and the window of implantation (WOI, 7–9 days post-ovulation). Based on 16S rRNA sequencing analysis, α-diversity showed no significant differences among the EFP, OP, and WOI in either vaginal (Chao1: *p* > 0.99; Shannon: *p* > 0.99) or cervical (Chao1: *p* > 0.99; Shannon: *p* > 0.99) microbiota. Likewise, β-diversity, assessed using Bray–Curtis distance, revealed no significant differences in vaginal (*p* > 0.50) or cervical (*p* > 0.50) communities (**Figure 1**). These results suggest a high degree of similarity in microbial composition between the vagina and cervix, with relative stability throughout the menstrual cycle. Consequently, subsequent analyses did not restrict the collection of vaginal and cervical swabs to specific menstrual phases, except for the exclusion of the menstrual period to minimize potential confounding effects.

### 3.2 LGT microbiota composition is associated with FET outcomes in PCOS patients

**Figure 2:**
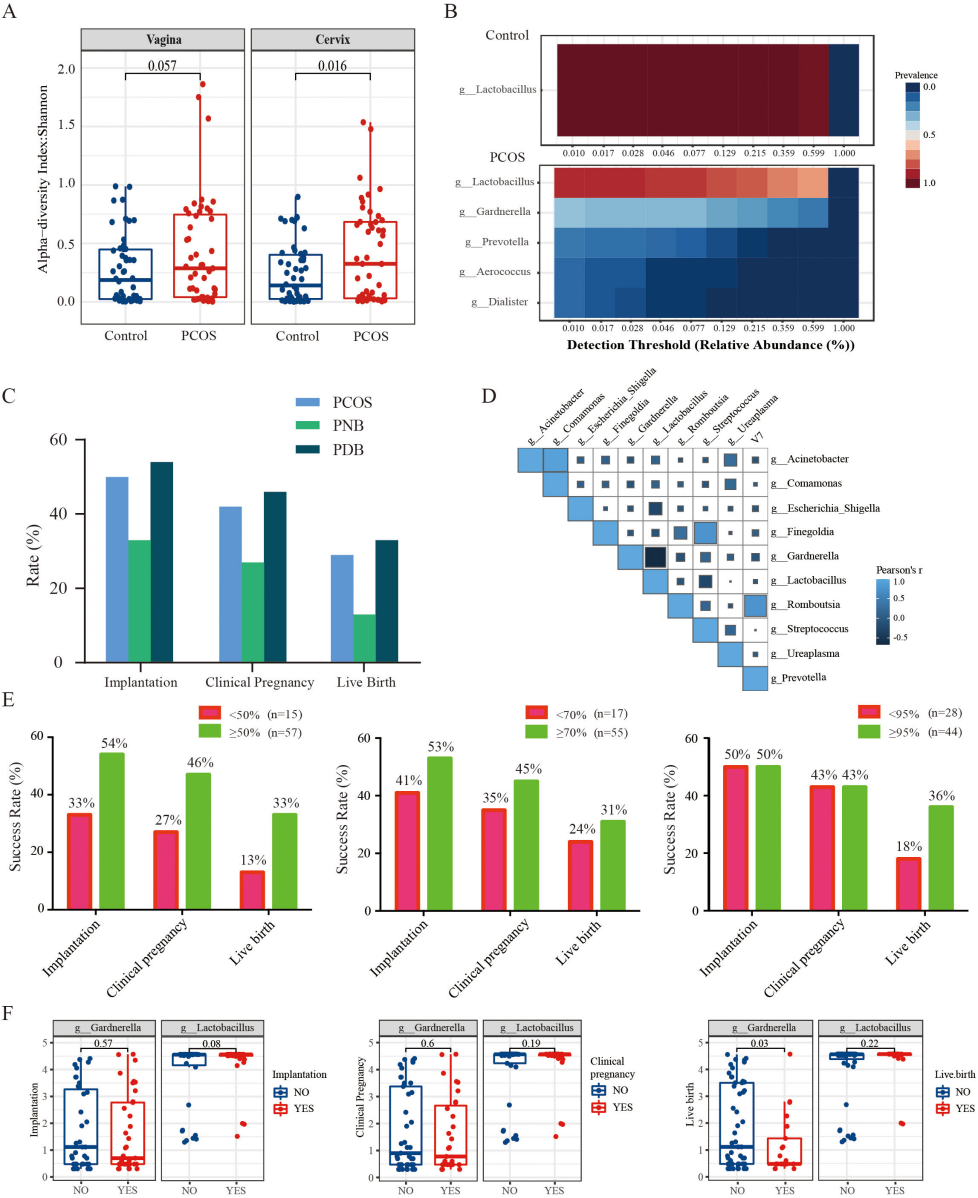
Composition of the LGT microbiota is associated with IVF-FET outcomes in patients with PCOS. **(A)** Alpha diversity analysis of the LGT microbiota in cervical and vaginal samples from control (n = 50) and PCOS (n = 47) groups, assessed using the Shannon index; **(B)** Heatmap showing the relative abundance of core vaginal microbial genera in control and PCOS groups; **(C)** Implantation, clinical pregnancy, and live birth rates among PCOS patients undergoing IVF-FET, including the total PCOS group (n = 72), PNB subgroup (n = 15), and PDB subgroup (n = 57); **(D)** Spearman correlation analysis of dominant microbial genera in the LGT microbiota of PCOS patients; **(E)** IVF-FET outcomes in PCOS patients stratified by vaginal *Lactobacillus* abundance: <50% vs. ≥50% (n = 15 vs. n = 57), <70% vs. ≥70% (n = 17 vs. n = 55), and <95% vs. ≥95% (n = 28 vs. n = 44); **(F)** Comparison of the relative abundance of *Gardnerella* and *Lactobacillus* across implantation, clinical pregnancy, and live birth outcomes; PNB, PCOS non-dependent on bacteria (*Lactobacillus* abundance <50%); PDB, PCOS dependent on bacteria (*Lactobacillus* abundance ≥50%).

Subsequently, the study examined compositional differences in the LGT microbiome between individuals with PCOS and healthy controls. Clinical characteristics of the 47 PCOS women and 50 healthy women were presented in **Supplementary Table 1**. The PCOS group exhibited elevated anti-Müllerian hormone (AMH) levels, an increased LH/FSH ratio, irregular menstrual cycles, and a trend toward higher testosterone levels, consistent with typical clinical features of PCOS. No statistically significant differences were observed between the two groups in other parameters, including age, body mass index (BMI), or abortion history. Analysis of the Shannon index revealed a significantly higher α-diversity in the PCOS group compared to the control group (**Figure 2A**). Notably, the LGT microbiota in the control group was predominantly composed of *Lactobacillus* species, whereas its relative abundance was significantly reduced in the PCOS group. Conversely, the PCOS group exhibited a marked enrichment of *Gardnerella*, *Prevotella*, *Aerococcus*, and *Dialister* species (**Figure 2B**).

To further investigate the potential impact of *Lactobacillus* abundance in LGT on clinical outcomes in patients with PCOS undergoing IVF-FET, an additional cohort of 72 PCOS participants were stratified based on their *Lactobacillus* content. Participants with <50% *Lactobacillus* abundance were defined as the PNB group (PCOS non-dependent on bacteria), while those with ≥50% were classified as the PDB group (PCOS dependent on bacteria). Detailed IVF-FET outcome for each group were presented in **Table 1**. The results showed that the PNB group exhibited significantly lower implantation, clinical pregnancy, and live birth rates compared to the PDB group (**Figures 2C, E**). Similarly, in patients with PCOS, a LGT *Lactobacillus* abundance exceeding 70% was associated with significantly higher implantation, clinical pregnancy, and live birth rates (**Figure 2E**). Although further increases in *Lactobacillus* abundance beyond 95% did not significantly affect implantation or clinical pregnancy rates, the live birth rate remained significantly higher than that in patients with *Lactobacillus* abundance below 95% (**Figure 2E**).

**Table 1:** Differences of FET outcomes between PDB and PNB group.

Group	PNB (n=15)	PDB (n=57)	PCOS (n=72)
Implantation Rate	0.33 (n=5) *	0.54 (n=31)	0.50 (n=36)
Clinical Pregnancy Rate	0.27 (n=4) *	0.46 (n=26)	0.42 (n=30)
Live Birth Rate	0.13 (n=2) *	0.33 (n=19)	0.29 (n=21)
Abortion Rate	0.60 (n=3)	0.39 (n=12)	0.42 (n=15)
Preterm Birth	0	(n=2)	(n=2)
Multiple Pregnancy	0	(n=2)	(n=2)
Mode of Delivery			
- Vaginal	0.50 (n=1)	0.21 (n=4)	0.24 (n=5)
- Cesarean	0.50 (n=1)	0.79 (n=15)	0.76 (n=16)
Age	30.4 ± 2.8	29.5 ± 3.4	29.8 ± 3.2

*P*-value was calculated using Fisher’s exact test, *p<0.05.

Correlation analysis of the LGT microbiota in patients with PCOS demonstrated a strong inverse association between the depletion of *Lactobacillus* and the enrichment of *Gardnerella* (**Figure 2D**). Additionally, comparative analysis revealed that *Gardnerella* abundance was significantly lower in PCOS patients who achieved live birth compared to those who did not (**Figure 2F**).

### 3.3 Testosterone are highly relevant to LGT microbiota dysbiosis in PCOS

**Figure 3:**
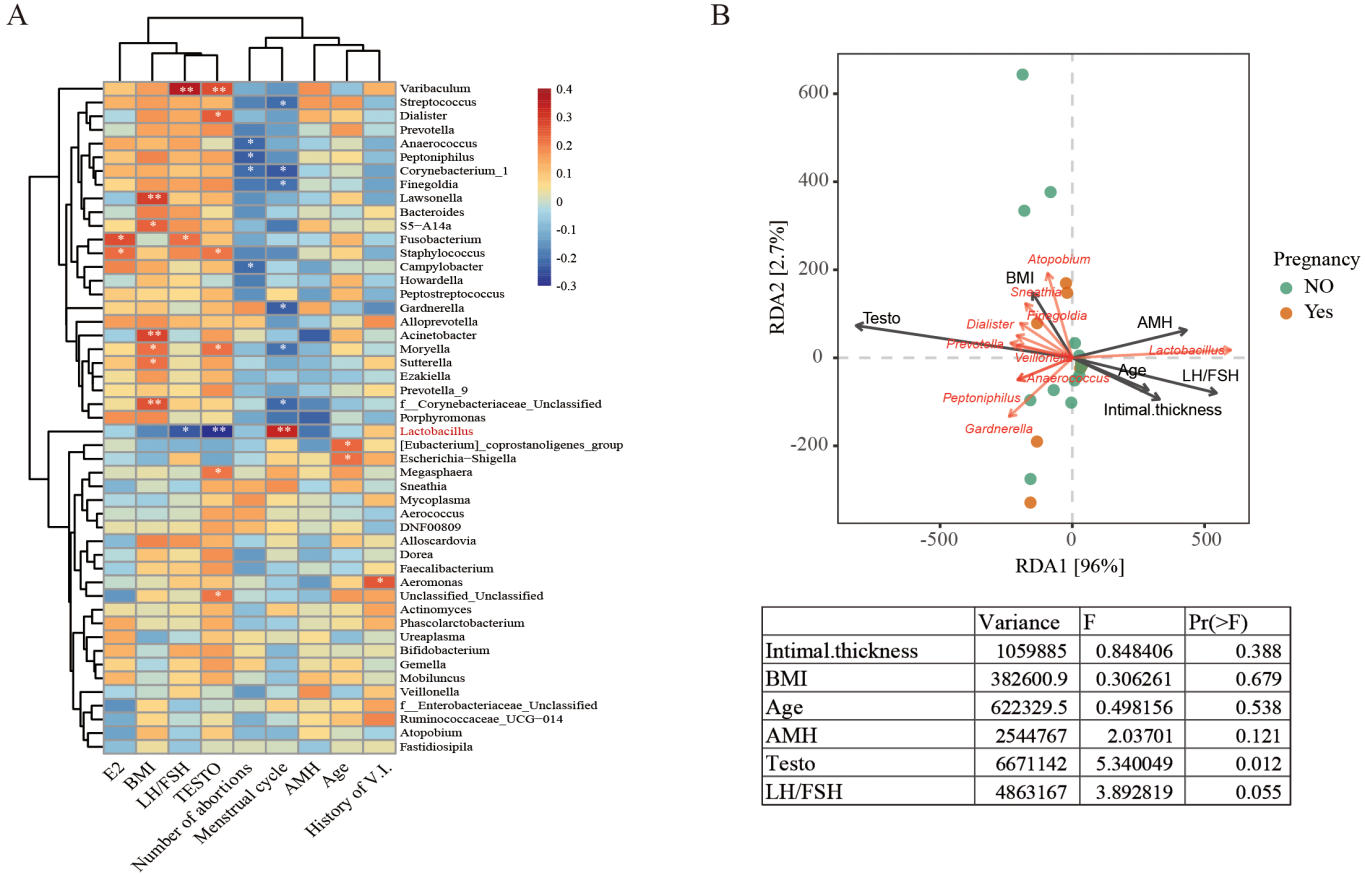
Associations between LGT microbiota composition and clinical parameters in PCOS patients undergoing IVF-FET. **(A)** Spearman correlation analysis between the relative abundance of LGT bacterial genera and clinical parameters. Red indicates a positive correlation, and blue indicates a negative correlation. *p < 0.05; **p < 0.01; **(B)** Redundancy analysis (RDA) depicting the relationships among dominant bacterial genera (red arrows), clinical parameters (black arrows), and pregnancy outcomes (orange and green dots). Arrow length reflects the strength of the association. The accompanying table summarizes the analysis of variance for clinical parameters, including variance, F-values, and *p*-values.

Subsequently, the potential associations between alterations in the LGT microbiome and clinical parameters were investigated. Spearman correlation analysis revealed that the relative abundance of *Lactobacillus* was positively correlated with menstrual cycle frequency (*p* < 0.01) and negatively correlated with serum testosterone level (*p* < 0.01) and LH/FSH ratio (*p* < 0.05) (**Figure 3A**). RDA analysis further demonstrated that the composition of the LGT microbiota in PCOS patients was strongly influenced by testosterone levels (**Figure 3B**). Elevated testosterone was associated with an increased abundance of non-dominant bacterial genera, such as *Gardnerella*, and a decreased abundance of *Lactobacillus*.

### 3.4 Alteration of LGT microbiota is related to changes in cervical epithelial cell function in PCOS patients

**Figure 4:**
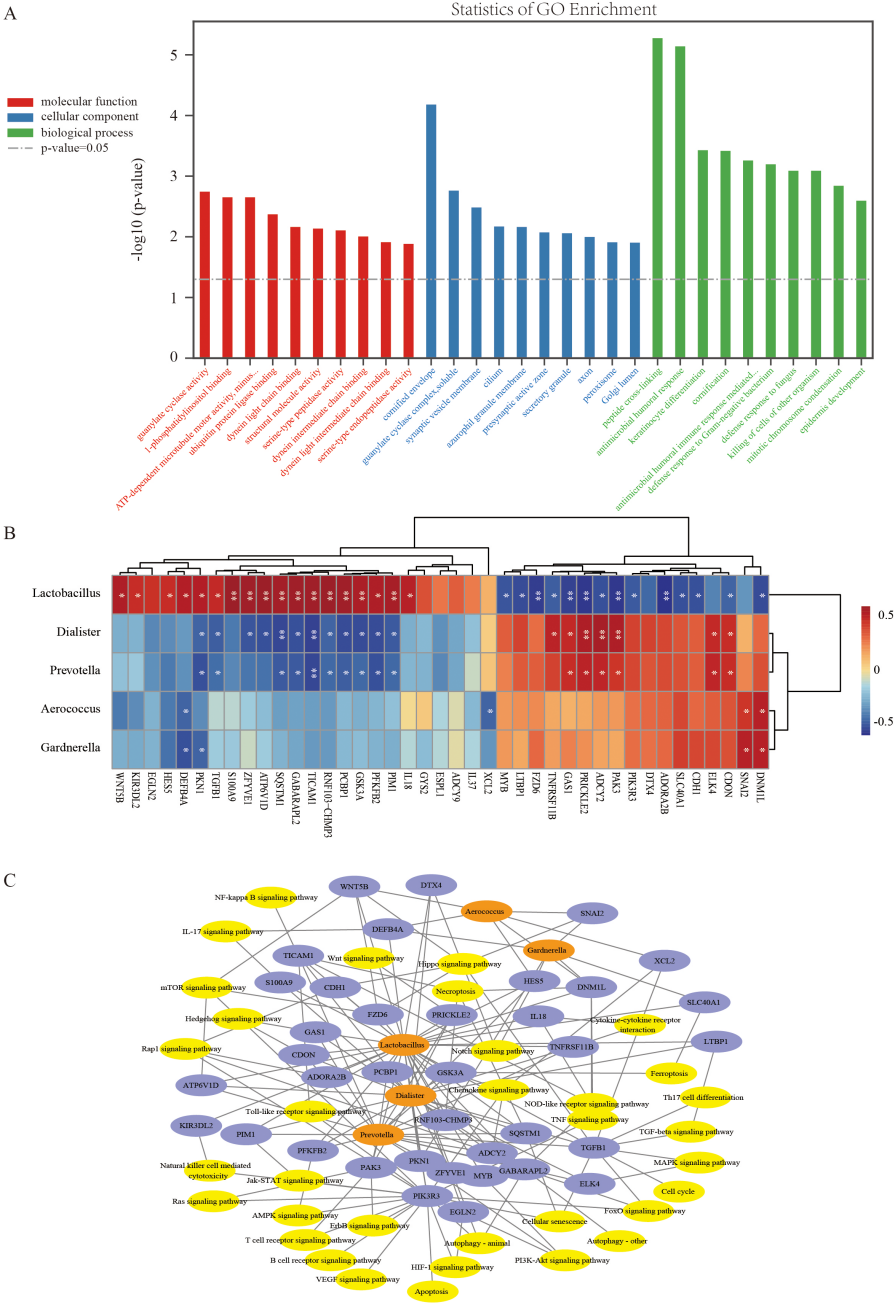
Alterations in the LGT microbiota are associated with functional changes in cervical epithelial cells in PCOS patients. **(A)** GO enrichment analysis of 497 DEGs in cervical epithelial cells between the PDB group (n = 5) and the PNB group (n = 4); **(B)** Correlation heatmap showing associations between DEGs involved in key signaling pathways and five representative bacterial genera. Red indicates positive correlations, and blue indicates negative correlations. *p < 0.05; **p < 0.01; **(C)** Network diagram illustrating the relationships among bacterial genera (orange), signaling pathways (yellow), and DEGs (blue). Edges represent correlations (absolute Spearman’s r > 0.6) or pathway associations. Microbiota–gene links indicate expression correlations, while gene–pathway links indicate pathway involvement.

To further investigate the potential association between alterations in the LGT microbiota and functional changes in mucosal epithelial cells in women with PCOS, gene expression profiles were compared between five patients from the PDB group and four from the PNB group. The corresponding clinical characteristics of these patients are summarized in **Supplementary Table 2**. A total of 497 differentially expressed genes (DEGs) were identified (*p* < 0.05). GO enrichment analysis revealed that these DEGs were primarily involved in biological processes such as peptide cross-linking, antimicrobial humoral responses, keratinocyte differentiation, cornification, and immune responses mediated by antimicrobial peptides (**Figure 4A**).

In addition, an association network was constructed to link the five major bacterial genera to the DEGs and relevant signaling pathways (**Figures 4B, C**). Notably, *Lactobacillus* was involved in key pathways, including the Notch and Toll-like receptor signaling pathways, and showed significant correlations with the expression of associated genes. These findings suggest that alterations in the composition of the LGT microbiota, particularly changes in *Lactobacillus* abundance, are closely associated with functional changes in the local mucosal epithelium.

## 4 Discussion

The stability of the reproductive microbiota throughout the menstrual cycle remains a subject of ongoing investigation (Alhabardi et al., 2021; Krog et al., 2022). In the present study, three distinct phases of the menstrual cycle—the early follicular phase, the ovulatory phase, and the window of implantation—were selected for 16S rRNA sequencing. The menstrual phase was excluded to avoid potential interference from menstrual blood and endometrial tissue shedding, which could compromise microbial detection. The results demonstrated that, despite substantial fluctuations in sex hormone levels, the composition of the LGT microbiota remained stable across these phases. These findings suggest that women with regular menstrual cycles maintain a consistent LGT microbiota throughout the cycle.

Extensive studies have highlighted a strong association between the composition of the genital tract microbiota and pregnancy outcomes in reproductive-aged women (Stout et al., 2017; Fettweis et al., 2019; Fu et al., 2020; Chen et al., 2022). For instance, a recent study by Fu et al. reported that women with recurrent implantation failure exhibited marked alterations in vaginal microbiota, particularly characterized by reduced *Lactobacillus* abundance (Fu et al., 2020). Similarly, a prospective cohort study found that a higher abundance of *Atopobium* in the LGT during early pregnancy was significantly associated with an increased risk of first-trimester spontaneous abortion (Chen et al., 2022). Additionally, multiple studies have linked *Lactobacillus* depletion to a heightened risk of spontaneous preterm birth (Stout et al., 2017; Fettweis et al., 2019). In line with these findings, the present study observed that PCOS patients with a relatively higher abundance of *Lactobacillus* in the LGT achieved more favorable IVF-FET outcomes, including higher implantation, clinical pregnancy, and live birth rates. Given that *Lactobacillus* species are typically dominant in the LGT of reproductive-aged women (Green et al., 2015; Chadchan et al., 2022), the protective role of a *Lactobacillus*-dominated microbiota—from implantation through parturition—appears to be well supported.

This study was the first to identify an inverse association between testosterone levels and the abundance of *Lactobacillus* in LGT. While previous studies have primarily focused on the impact of androgens on the gut microbiome, animal models have demonstrated that androgen deprivation through castration induces a shift in the male gut microbial profile toward that of females. Conversely, testosterone supplementation in gonadectomized males has been shown to reverse this shift (Yurkovetskiy et al., 2013; Org et al., 2016). In humans, women with PCOS frequently exhibit gut microbial dysbiosis (Insenser et al., 2018; Zhou et al., 2020). Mechanistically, testosterone may influence the gut microbiota through pathways such as bile acid metabolism (Org et al., 2016); however, its impact on the LGT microbiome remains largely unexplored and requires further investigation.

The association between *Lactobacillus* abundance in the LGT microbiota and IVF-FET outcomes in PCOS patients may be attributed to its role in maintaining a healthy cervical and vaginal microenvironment. Previous studies have demonstrated that the relative abundance of *Lactobacillus* in the genital tract is closely associated with the integrity and function of cervicovaginal mucus, while *Lactobacillus* deficiency has been linked to impaired immune homeostasis (Doerflinger et al., 2014; Anahtar et al., 2015; Lacroix et al., 2020; Mohd Zaki et al., 2022). Furthermore, vaginal administration of *Lactobacillus crispatus* following standard treatment for bacterial vaginosis has been shown to result in a sustained reduction in genital inflammation and improvements in biomarkers of epithelial integrity (Armstrong et al., 2022). In the present study, several DEGs between the PDB and PNB groups were identified, including *TICAM3, PKN1, TGFB1, GSK3A*, and *SQSTM1*, which were downregulated in the PNB group, and *PAK3, PRICKLE2*, and *GAS1*, which exhibited higher expression in the PNB group. Pathway enrichment analysis indicated that these genes are involved in biological processes such as the antimicrobial humoral response, defense response to fungi, and cytotoxic activity against other organisms. These findings suggest that women with PCOS and a relatively lower abundance of *Lactobacillus* in the LGT may be more susceptible to an immune-imbalanced microenvironment. Additionally, significant alterations were observed in pathways related to keratinocyte differentiation between the two groups, indicating that epithelial barrier formation may be compromised. Collectively, these results suggest that the LGT microbiota in women with PCOS plays a critical role in maintaining local mucosal epithelial barrier integrity and immune homeostasis.

## 5 Conclusion

In conclusion, the findings of this study suggests that dysbiosis of the LGT microbiota in women with PCOS, particularly the depletion of *Lactobacillus*, is associated with poorer FET outcomes and may potentially impair the function of cervical epithelial cells. Further research is warranted to elucidate the underlying mechanisms governing the interaction between the LGT microbiota and the reproductive system in PCOS patients. A deeper understanding of these relationships could provide novel insights and inform clinical strategies for diagnosis and treatment.

## Data Availability

All sequence data are available at the National Center for Biotechnology Information. The 16S rRNA sequences were deposited under accession numbers SAMN21619565-21619724, SAMN21619392-21619411, SAMN21619461-21619544, SAMN21619730-21619738. RNA-Seq data are deposited under BioProject ID PRJNA766136, SRA accession numbers SUB10427247-10428494.

## References

[B1] Abou Chacra L. Fenollar F. (2021). Exploring the global vaginal microbiome and its impact on human health. Microb. Pathog. 160, 105172. doi: 10.1016/j.micpath.2021.105172, PMID: 34500016

[B2] Alhabardi S. M. Edris S. Bahieldin A. Al-Hindi R. R. (2021). The composition and stability of the vaginal microbiome of healthy women. J. Pak Med. Assoc. 71, 2045–2051. doi: 10.47391/JPMA.1465, PMID: 34418027

[B3] Anahtar M. N. Byrne E. H. Doherty K. E. Bowman B. A. Yamamoto H. S. Soumillon M. . (2015). Cervicovaginal bacteria are a major modulator of host inflammatory responses in the female genital tract. Immunity 42, 965–976. doi: 10.1016/j.immuni.2015.04.019, PMID: 25992865 PMC4461369

[B4] Armstrong E. Hemmerling A. Miller S. Burke K. E. Newmann S. J. Morris S. R. . (2022). Sustained effect of LACTIN-V (Lactobacillus crispatus CTV-05) on genital immunology following standard bacterial vaginosis treatment: results from a randomised, placebo-controlled trial. Lancet Microbe 3, e435–ee42. doi: 10.1016/S2666-5247(22)00043-X, PMID: 35659905 PMC9188188

[B5] Auriemma R. S. Scairati R. Del Vecchio G. Liccardi A. Verde N. Pirchio R. . (2021). The vaginal microbiome: A long urogenital colonization throughout woman life. Front. Cell Infect. Microbiol. 11, 686167. doi: 10.3389/fcimb.2021.686167, PMID: 34295836 PMC8290858

[B6] Chadchan S. B. Singh V. Kommagani R. (2022). Female reproductive dysfunctions and the gut microbiota. J. Mol. endocrinol 69, R81–R94. doi: 10.1530/JME-21-0238, PMID: 35900833 PMC10031513

[B7] Chen S. Xue X. Zhang Y. Zhang H. Huang X. Chen X. . (2022). Vaginal atopobium is associated with spontaneous abortion in the first trimester: a prospective cohort study in China. Microbiol. Spectr. 10, e0203921. doi: 10.1128/spectrum.02039-21, PMID: 35311570 PMC9045190

[B8] Colella M. Topi S. Palmirotta R. D’Agostino D. Charitos I. A. Lovero R. . (2023). An overview of the microbiota of the human urinary tract in health and disease: current issues and perspectives. Life (Basel) 13, 1486. doi: 10.3390/life13071486, PMID: 37511861 PMC10381901

[B9] Doerflinger S. Y. Throop A. L. Herbst-Kralovetz M. M. (2014). Bacteria in the vaginal microbiome alter the innate immune response and barrier properties of the human vaginal epithelia in a species-specific manner. J. Infect. Dis. 209, 1989–1999. doi: 10.1093/infdis/jiu004, PMID: 24403560

[B10] Elovitz M. A. Gajer P. Riis V. Brown A. G. Humphrys M. S. Holm J. B. . (2019). Cervicovaginal microbiota and local immune response modulate the risk of spontaneous preterm delivery. Nat. Commun. 10, 1305. doi: 10.1038/s41467-019-09285-9, PMID: 30899005 PMC6428888

[B11] Fettweis J. M. Serrano M. G. Brooks J. P. Edwards D. J. Girerd P. H. Parikh H. I. . (2019). The vaginal microbiome and preterm birth. Nat. Med. 25, 1012–1021. doi: 10.1038/s41591-019-0450-2, PMID: 31142849 PMC6750801

[B12] Fu M. Zhang X. Liang Y. Lin S. Qian W. Fan S. (2020). Alterations in vaginal microbiota and associated metabolome in women with recurrent implantation failure. mBio 11, e03242-19. doi: 10.1128/mBio.03242-19, PMID: 32487762 PMC7267891

[B13] Graham M. E. Herbert W. G. Song S. D. Raman H. N. Zhu J. E. Gonzalez P. E. . (2021). Gut and vaginal microbiomes on steroids: implications for women’s health. Trends Endocrinol. metabolism: TEM 32, 554–565. doi: 10.1016/j.tem.2021.04.014, PMID: 34049772 PMC8282721

[B14] Green K. A. Zarek S. M. Catherino W. H. (2015). Gynecologic health and disease in relation to the microbiome of the female reproductive tract. Fertility sterility 104, 1351–1357. doi: 10.1016/j.fertnstert.2015.10.010, PMID: 26597627

[B15] Hoeger K. M. Dokras A. Piltonen T. (2021). Update on PCOS: consequences, challenges, and guiding treatment. J. Clin. Endocrinol. Metab. 106, e1071–e1e83. doi: 10.1210/clinem/dgaa839, PMID: 33211867

[B16] Insenser M. Murri M. Del Campo R. Martinez-Garcia M. A. Fernandez-Duran E. Escobar-Morreale H. F. (2018). Gut microbiota and the polycystic ovary syndrome: influence of sex, sex hormones, and obesity. J. Clin. Endocrinol. Metab. 103, 2552–2562. doi: 10.1210/jc.2017-02799, PMID: 29897462

[B17] Krog M. C. Hugerth L. W. Fransson E. Bashir Z. Nyboe Andersen A. Edfeldt G. . (2022). The healthy female microbiome across body sites: effect of hormonal contraceptives and the menstrual cycle. Hum. reproduction 37, 1525–1543. doi: 10.1093/humrep/deac094, PMID: 35553675 PMC9247429

[B18] Lacroix G. Gouyer V. Gottrand F. Desseyn J. L. (2020). The cervicovaginal mucus barrier. Int. J. Mol. Sci. 21, 8266. doi: 10.3390/ijms21218266, PMID: 33158227 PMC7663572

[B19] Liao B. Qiao J. Pang Y. (2021). Central regulation of PCOS: abnormal neuronal-reproductive-metabolic circuits in PCOS pathophysiology. Front. endocrinol. 12, 667422. doi: 10.3389/fendo.2021.667422, PMID: 34122341 PMC8194358

[B20] Mei Z. Li D. (2022). The role of probiotics in vaginal health. Front. Cell Infect. Microbiol. 12, 963868. doi: 10.3389/fcimb.2022.963868, PMID: 35967876 PMC9366906

[B21] Mohd Zaki A. Hadingham A. Flaviani F. Haque Y. Mi J. D. Finucane D. . (2022). Neutrophils dominate the cervical immune cell population in pregnancy and their transcriptome correlates with the microbial vaginal environment. Front. Microbiol. 13, 904451. doi: 10.3389/fmicb.2022.904451, PMID: 35774454 PMC9237529

[B22] Org E. Mehrabian M. Parks B. W. Shipkova P. Liu X. Drake T. A. . (2016). Sex differences and hormonal effects on gut microbiota composition in mice. Gut Microbes 7, 313–322. doi: 10.1080/19490976.2016.1203502, PMID: 27355107 PMC4988450

[B23] Patel S. (2018). Polycystic ovary syndrome (PCOS), an inflammatory, systemic, lifestyle endocrinopathy. J. Steroid Biochem. Mol. Biol. 182, 27–36. doi: 10.1016/j.jsbmb.2018.04.008, PMID: 29678491

[B24] Peelen M. J. Luef B. M. Lamont R. F. de Milliano I. Jensen J. S. Limpens J. . (2019). The influence of the vaginal microbiota on preterm birth: A systematic review and recommendations for a minimum dataset for future research. Placenta 79, 30–39. doi: 10.1016/j.placenta.2019.03.011, PMID: 31047708

[B25] Stout M. J. Zhou Y. Wylie K. M. Tarr P. I. Macones G. A. Tuuli M. G. (2017). Early pregnancy vaginal microbiome trends and preterm birth. Am. J. obstetrics gynecol 217, 356 e1–35 e18. doi: 10.1016/j.ajog.2017.05.030, PMID: 28549981 PMC5581228

[B26] Torcia M. G. (2019). Interplay among vaginal microbiome, immune response and sexually transmitted viral infections. Int. J. Mol. Sci. 20, 266. doi: 10.3390/ijms20020266, PMID: 30641869 PMC6359169

[B27] Tu Y. Zheng G. Ding G. Wu Y. Xi J. Ge Y. . (2020). Comparative analysis of lower genital tract microbiome between PCOS and healthy women. Front. Physiol. 11, 1108. doi: 10.3389/fphys.2020.01108, PMID: 33013474 PMC7506141

[B28] Wang S. Zhao H. Li F. Xu Y. Bao H. Zhao D. (2022). Higher chronic endometritis incidences within infertile polycystic ovary syndrome clinical cases. J. Healthc Eng 2022, 9748041. doi: 10.1155/2022/9748041, PMID: 35449841 PMC9017445

[B29] Yurkovetskiy L. Burrows M. Khan A. A. Graham L. Volchkov P. Becker L. . (2013). Gender bias in autoimmunity is influenced by microbiota. Immunity 39, 400–412. doi: 10.1016/j.immuni.2013.08.013, PMID: 23973225 PMC3822899

[B30] Zhou L. Ni Z. Yu J. Cheng W. Cai Z. Yu C. (2020). Correlation between fecal metabolomics and gut microbiota in obesity and polycystic ovary syndrome. Front. endocrinol 11, 628. doi: 10.3389/fendo.2020.00628, PMID: 33013704 PMC7505924

