# Supplementary material for: The composition of lower genital tract microbiota correlates with in vitro fertilization and frozen embryo transfer outcomes in women with polycystic ovarian syndrome
Source: Front Cell Infect Microbiol. 2025 Dec 8;15:1617187. doi: 10.3389/fcimb.2025.1617187 (PMC12719300; doi:10.3389/fcimb.2025.1617187)
Supplement: Supplementary file 1 [file Table1.docx]

Supplementary Table 1: Clinical characteristics of reproductive-aged healthy women and PCOS women

| Group |  | Control women (n=50) | PCOS women (n=47) | *p* value |
| --- | --- | --- | --- | --- |
| Age |  | 31.4±4.0 | 29.2±4.0 | 0.711 |
| BMI |  | 22.1±3.0 | 24.2±3.9 | 0.134 |
| Testo |  | 1.6±0.6 | 2.3±0.7 | 0.067 |
| AMH |  | 7.1±4,3 | 10.3±4,3 | 0.003 |
| E2 |  | 151.8±64.4 | 189.0±90.3 | 0.102 |
| LH/FSH |  | 0.7±0.4 | 1.2±0.8 | 0.002 |
| Vaginosis History | Yes | n=21 | n=3 | 0.012 |
|  | No | n=29 | n=44 |  |
| Abortion History | Yes | n=19 | n=21 | 0.205 |
|  | No | n=31 | n=26 |  |
| Menstrual cycles / per year |  | 12.4±1.1 | 5.8±1.7 | 0.005 |
