# Supplementary material for: The composition of lower genital tract microbiota correlates with in vitro fertilization and frozen embryo transfer outcomes in women with polycystic ovarian syndrome
Source: Front Cell Infect Microbiol. 2025 Dec 8;15:1617187. doi: 10.3389/fcimb.2025.1617187 (PMC12719300; doi:10.3389/fcimb.2025.1617187)
Supplement: Supplementary file 2 [file Table2.docx]

Supplementary Table 2: Clinical Characteristics of Women in the PDB and PNB Groups

| Group | PNB (n=4) | PDB (n=5) | *P* value |
| --- | --- | --- | --- |
| Age | 26.3±0.9 | 28.2±3.7 | ns |
| PCO | 4 | 5 |  |
| Menstrual cycles / per year | 7.0±2.0 | 7.8±3.1 | ns |
| BMI | 21.7±5.2 | 25.5±1.7 | * |
| Testo | 1.8±0.2 | 2.9±0.6 | * |

ns: no significant; **p*<0.05
